# Why Do Ionic Surfactants Significantly Alter the Chemiluminogenic Properties of Acridinium Salt?

**DOI:** 10.3390/molecules29163736

**Published:** 2024-08-07

**Authors:** Magdalena Mańkowska, Karol Krzymiński, Dariusz Wyrzykowski, Beata Zadykowicz, Sergey A. Samsonov

**Affiliations:** Faculty of Chemistry, University of Gdańsk, Wita Stwosza 63, 80-308 Gdansk, Poland; m.mankowska.647@studms.ug.edu.pl (M.M.); dariusz.wyrzykowski@ug.edu.pl (D.W.); beata.zadykowicz@ug.edu.pl (B.Z.)

**Keywords:** chemiluminescence, ionic surfactants, critical micelle concentration, acridinium esters, reaction mechanism, molecular dynamics

## Abstract

Acridinium esters, due to their capability for chemiluminescence (CL), are employed as indicators and labels in biomedical diagnostics and other fields. In this work, the influence of ionic surfactants, hexadecyltrimethylammonium chloride and bromide (CTAC and CTAB, cationic) and sodium dodecyl sulphate (SDS, anionic) on the CL parameters and mechanism of representative emitter, 10-methyl-9-[(2-methylphenoxy)carbonyl]acridinium trifluoromethanesulphonate (2MeX) in a H_2_O_2_/NaOH environment, is studied. Our investigations revealed that the type of surfactant and its form in solution have an impact on the CL kinetic constants and integral efficiencies, while changes in those emission properties resulting from the type of ion (Cl^−^ vs. Br^−^) are negligible. The major changes were recorded for systems containing surfactants at concentrations higher than the critical micelle concentration. The cationic surfactants (CTAC, CTAB) cause a substantial increase in CL emission kinetics and a moderate increase in its integral efficiency. At the same time, the opposite effect is observed in the case of SDS. Molecular dynamics simulations suggest that changes in emission parameters are likely due to differences in the binding strength of 2MeX substrate with surfactant molecules, which is higher for SDS than for CTAC. The results can help in rational designing of optimal acridinium CL systems and demonstrate their usefulness in distinguishing the pre- and post-micellar environment and the charge of surfactants.

## 1. Introduction

Acridinium aromatic esters (AEs) belong to the systems capable of efficient chemical transformation into electronically excited products, i.e., chemiluminescence (CL) [1,2]. Unlike popular chemiluminogens such as derivatives of luminol, the main advantages of AEs are the relatively high quantum yield of emission with a low background signal and not needing the use of a catalyst for initiating CL, as well as relatively quick and easy control of its dynamics [3,4]. All these aspects have rendered AEs broadly employed as CL labels or indicators in medical/pharmaceutical [5,6], chemical and biochemical [7,8], and environmental and food analytics today [9]. CL labels based on them have been used to determine the concentration of hormones [10,11], antibodies and antigens [12,13,14], enzymes [15], antioxidants [4,16] and nucleic acid fragments [17] at an ultra-sensitive level (limits of detection at the level of 10^−19^ mole of analyte or below [18]).

**Figure 1:**
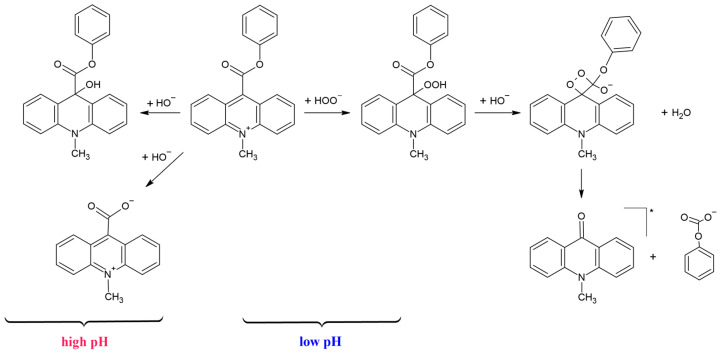
Proposed chemiluminescence mechanism of acridinium esters [19,20].

Our previous computational studies have indicated that AEs readily undergo oxidation in the presence of hydrogen peroxide in alkaline media, initiating the emission of light (Figure 1) [19,20]. In the first step of this process, the OOH^−^ ions, formed from the oxidant H_2_O_2_ in an alkaline environment, attack the electrophilic centre of AE, i.e., the carbon atom in position 9 of the acridinium moiety (the C atom with the highest electron deficit [19,20]). Next, due to bond rearrangements, a cyclic intermediate product is formed: the acridane dioxethanone derivative. Its decarboxylation occurs, and an electronically excited product, 10 substituted-acridan-9-one, is formed. Returning to the ground state, the latter emits radiation in the visible range (ca. 450 nm in water).

One of the key parameters that significantly influences the efficiency of the emission of light of AEs is the appropriate acid–base equilibrium of its aqueous solution before the initiation of CL (Figure 1). At a high pH, so-called pseudobases of AE are formed [20]. The latter reacts slowly with OOH^−^ ions, but this process does not produce electronically excited CL products [19,20]. In an alkaline environment, AEs can undergo hydrolysis to form 10 substituted-9-carboxy acridinium acid salt [20]. At low pH, the acid–base equilibrium is shifted towards the acridinium cation. The effectiveness of CL is higher when the cationic form of AE is at the maximum level in the system. Therefore, slightly acidic conditions must be maintained before the CL emission is started.

One way to increase the efficiency of the CL process is to use organised media, which can play a significant role by accelerating the transformation’s kinetics and increasing the emission efficiency, resulting in higher sensitivity of luminometric measurements [21].

Organised media are widely employed in chemical synthesis and analysis to modify the solubility and environment, appropriately orient solute molecules of a bulk solution, enhance reactivity and control the course of chemical or photochemical transformations and biological processes [22,23,24,25,26]. The two types of organised media were researched to improve the CL efficiency of chemiluminogenic acridinium salts [21]. These were micellar media formed by surfactants, which, at specific concentrations exceeding the critical micelle concentration (CMC), combine forming aggregates termed micelles and cyclodextrins consisting of a macro-cyclic structure of glucose subunits joined by α-1,4-glycosidic bonds. Surfactants can be divided into four main types depending on their charge and structure: cationic, anionic, amphoteric and nonionic [22]. Their specific physicochemical properties, such as their propensity to undergo self-aggregation processes leading to micelle formation, are related to their ability to interact between the hydrophobic and hydrophilic parts of the solute molecule [23]. The equilibrium between micelles and monomer molecules can be affected by external factors, leading to changes in micelle stability and, thus, changes in the properties of a given system. The main factors influencing the CMC of surfactants include pH, temperature, ionic strength, solvent properties, electrolytes or non-electrolytes, and the technique used to measure the CMC [27,28,29]. Therefore, from a practical point of view, it is essential to control the form (monomers or micelles) in which the surfactant molecules are present in the system under investigation. According to that, the determination of the CMC is a widely researched topic today, and various methods are available for identifying the micellar environment [30].

In the 1980s, Grayeski and co-workers [31,32] reported that the efficiency of the CL reaction of acridine derivatives was increased by adding cyclodextrins as well as surfactants such as cetyltrimethylammonium chloride (CTAC) to the solution. The latter is the most commonly used micellar medium, which reduces the emission time of acridinium CL to less than 5 s and substantially increases its efficiency [33,34,35]. Natrajan’s group, investigating various types of surfactants, suggested that they affect light emission from AEs through two general mechanisms, depending on their charge and the influence of their micelles on mono- or bimolecular types of transformations [33]. The studies indicated that the maximum surfactant effect is obtained for the relatively hydrophobic acridinium moiety. However, it was also noticed that hydrophilic functional groups (in particular, sulfobetaine zwitterions) substituted in acridinium moiety in the presence of CTAC reduced the emission time while increasing the CL efficiency. The most significant improvement in the CL parameters of acridinium derivatives was achieved when the hydrophobic acridinium ring was combined with a hydrophilic leaving (phenyl) group [34]. The same group of researchers obtained interesting results by investigating the CL properties of fluorinated acridinium labels (AL) in the presence of cationic (CTAC) and anionic agents (sodium perfluorooctanoate, SPFO) and their mixtures. The studies indicated that the mixed system (CTAC and SPFO) at low mole fractions of SPFO leads to enhanced emission [36]. The authors suggested that enhancement in AL light yield is sensitive to the polarity of the micellar interface. 

However, it should be considered that the mentioned agents (surfactants) can be toxic, especially to aquatic organisms, when released into the environment. In this context, degradable cationic surfactants containing amide and carbonate groups can effectively substitute classical surfactants. In particular, the carbonate surfactant (dodecyl-3-(trimethylazane)propyl carbonate chloride) is especially promising because it mimics the behaviour of CTAC in affecting the CL of AE. The latter substance has limited stability in the final (post-reaction) mixture but is characterised by excellent long-term stability in acidic solutions. Therefore, it could effectively replace CTAC in systems to enhance the CL of AEs in automated immunoassays, being a more environmentally friendly alternative to CTAC [37].

Although the noticeable effect of surfactants on improving the CL efficiency of AEs is demonstrated in many experimental studies [20,31,32,33,34,35,36,37], there are no reports explaining at the molecular level why surfactants significantly alter the emissive properties of acridinium salts. The answer to the last question became the motive to undertake the proposed research experimentally and computationally. Molecular dynamics (MD)-based approaches can help characterise the potential interactions of surfactant molecules with intermediate products formed during the transformation of AEs in the process of their chemiluminogenic oxidation. To our knowledge, no computational studies have been conducted so far providing potential explanations for the atomistic mechanism of interaction of AEs with surfactants and their influence on the parameters of the CL process, which renders such an attempt very promising. In this work, we present a relatively simple chemiluminogenic compound—acridinium alkyl phenyl ester—as a representative and effective indicator to identify micellar systems and to distinguish the charge of micelle surfaces. The interactions between AE molecules and product(s) of its transformations with ionic surfactants may thus be a diagnostic tool for determining specific features of luminogenic systems, such as pre- or post-micellar concentration ranges, type of surfactant introduced, etc. The latter can be utilised in designing new CL systems with beneficial features potentially useful in various fields, such as pharmaceutical, biotechnology or materials science.

## 2. Results and Discussion

### 2.1. Critical Micellar Concentrations of Surfactants in CL Systems 

**Figure 2:**
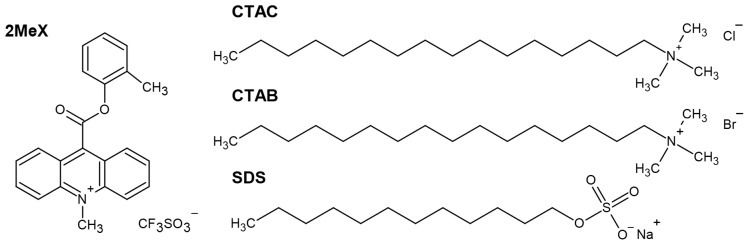
Structural formula of compounds investigated in this work: 10-methyl-9-[(2-methylphenoxy)carbonyl]-acridinium trifluoromethanesulphonate (2MeX)—chemiluminogenic substrate; CTAC, CTAB and SDS—surfactants.

Conductometric measurements were carried out to determine the critical micellar concentrations (CMC) of the surfactants employed in this work (Figure 2): CTAC, CTAB and SDS. The experiments were carried out at the conditions of CL measurements, before triggering the emission of light.

Titration curves express the dependence of solutions’ conductivity on the actual concentration of surfactant and are shown in Figure S1 in Supplementary Materials. The presence of strong electrolytes such as HCl and HNO_3_ (at mM levels of concentration) results in an increase in the ionic strength of the solution, which is manifested by changes in the values of their CMC in the system. The determined CMC at such conditions is slightly lower than in water, by approximately 9–34%, depending on the surfactant (Table 1).

### 2.2. Chemiluminogenic Properties of Acridinium Salt–Surfactant Systems

**Figure 3:**
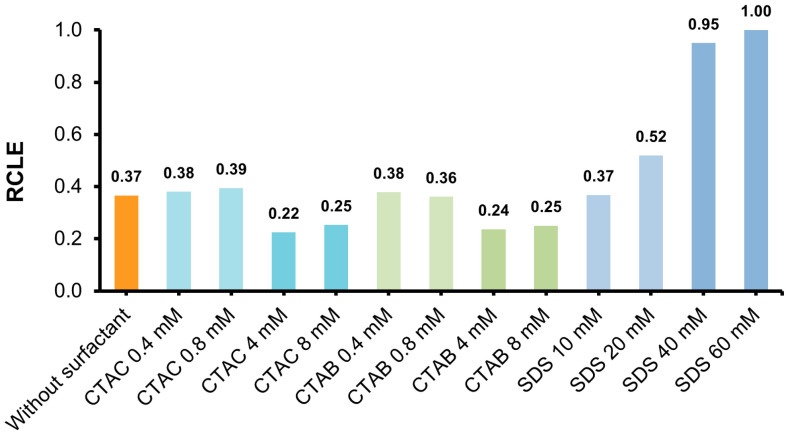
Comparison of the relative chemiluminescence efficiencies (RCLEs, normalised to the highest value) of 2MeX in the presence of various ionic surfactants. RSD values assessed for the parameters obtained in measurements fall in the range of 0.8–6.7%; for details, see Section 3.

**Figure 4:**
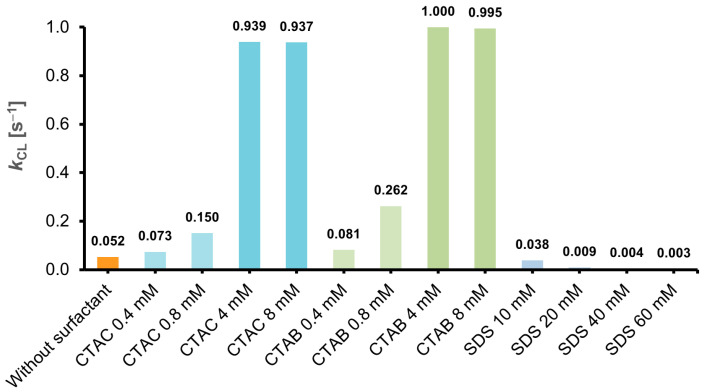
Comparison of the kinetics constants of CL decay (*k*_CL_, normalised to the highest value) of 2MeX in the presence of ionic surfactants. RSD values assessed for the parameters obtained in measurements fall in the range of 0.8–6.7%, for details see Section 3.

In order to determine the influence of ionic surfactants (CTAC, CTAB and SDS) on the emission parameters (kinetics and emission intensity of CL) of the investigated acridinium salt (2MeX, Figure 2), measurements of CL were performed, applying a plate format, optimal for this technique. Depending on the type and concentration of the surfactant investigated, a completely different effect on the parameters mentioned above was observed (Figure 2, Figure 3 and Figure 4). Detailed experimental data obtained from measurements of CL for all the systems mentioned above (maximum emission intensity values, *I*_CL_, max; emission decay rate constants, *k*_CL_; and integral emission efficiencies, RCLE) are shown in Table 2.

The chemiluminogenic system containing no surfactant (2MeX/H_2_O_2_/NaOH) is characterized by moderate kinetics and relatively good intensity of emission, compared to other compounds capable of CL (the quantum yield of 2MeX in water is 1.60% [19], and 1.23% for luminol [38]). The addition of cationic surfactants (CTAC or CTAB) to the investigated systems is manifested by a significant increase in the intensity of temporary CL and a shortening of the emission time and in result, a typical flash kinetics appears (Figures S2 and S3 in Supplementary Materials). This increase is smaller at the pre-micellar concentration and greater after reaching CMC, depending on their content in the system and slightly on the type of counter ion, involved in the surfactant’s molecular system (Cl^−^ vs. Br^−^) (Figure 2). On the other hand, the introduction of an anionic surfactant (SDS) causes a dramatic decrease in the temporary CL intensity and, resultantly, a significant increase in the emission time, which is manifested by the appearance of typical glow-type kinetics (Figures S3 and S4 in Supplementary Materials).

**Figure 5:**
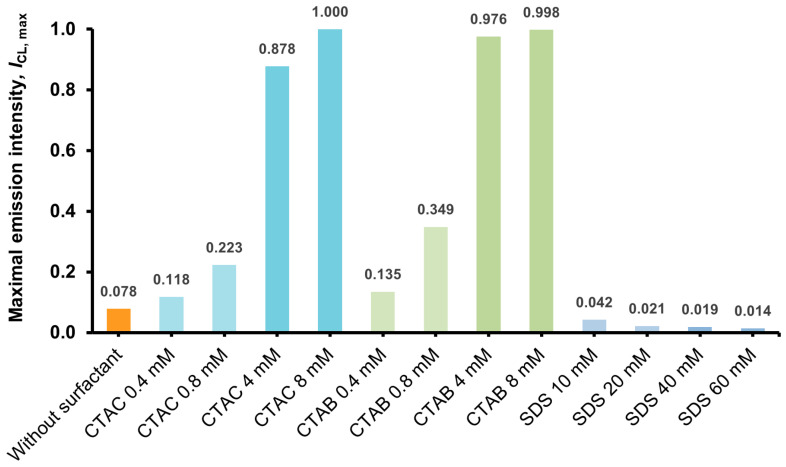
Comparison of the maximal values of CL intensity (*I*_CL_, max, normalised to the highest value) of 2MeX in the presence of various ionic surfactants (RSD = 0.8–6.7%). For details, see Section 3.

Treating the CL emission decays as pseudo-first-order processes, the kinetic constants of the investigated systems were determined, applying a graphical method [39] (Figures S6–S9 in Supplementary Materials). The values of the CL emission decay rate constants (*k*_CL_), as well as the intensities of maximum CL emission (*I*_CL_, max), increase significantly in the presence of cationic surfactants CTAC and CTAB and decrease in the presence of anionic SDS (Figure 3, Figure 4 and Figure 5). The determined values of *k*_CL_ correlate well with the *I*_CL_, max values (Figure S10 in Supplementary Materials). According to the abovementioned and considering the dynamics of light emission characterising acridinium CL, significant differences can be observed, depending on the type of surfactant introduced into the CL systems, as well as their concentration (pre- vs. post-micellar form).

Interestingly, when comparing the relative chemiluminescence efficiencies (integrals under reaction profiles, RCLE), different behaviour was observed compared to the emission dynamics changes. Namely, the presence of cationic surfactants (CTAC and CTAB) in the micellar form (>CMC) causes the RCLE value to decrease by almost half and increase by almost three times in the presence of SDS. In turn, for systems containing the investigated surfactants at the levels of pre-micellar concentration (<CMC), no significant changes in this parameter were observed compared to the system without their addition (Figure 3).

The CL parameters varied with the type of surfactant (cationic/anionic) and the pre-and post-micellar environment. Cationic surfactants exert a pronounced influence on the emission dynamics of CL, with a moderate impact on its efficiency. In contrast, the anionic surfactant exhibited an opposite effect, significantly slowing the emission dynamics and enhancing efficiency. The CL kinetic constants and integral efficiencies in the presence of ionic surfactants were assessed before and after the CMC points and determined conductmetrically at comparable conditions. It has been observed that near and above the point of CMC, there is a significant (ca. 18-fold) increase in the emission rate constant for CTAC and a ca. 15-fold decrease in this parameter for SDS. The observed effects of surfactant type and environment (pre- and post-micellar concentrations of surfactants) on the CL of the studied acridinium salt were quantified and compared in Table 3.

### 2.3. Ability to Fluorescence of Post-CL Mixtures Containing Surfactants

Fluorescence (FL) emission measurements were performed to consider the possible influence of ionic surfactants on emissive features of 10-methylacridan-9-one (10Me-Aon): the main product contained in post-CL reaction mixtures. The latter compound is a highly fluorescent molecule, the target emitter produced upon chemiluminogenic oxidation of AE with the NaOH/H_2_O_2_ system [19]. FL measurements were performed with the concentrations of studied surfactants above the CMC because of the most pronounced changes observed in such conditions, as was mentioned above.

**Figure 6:**
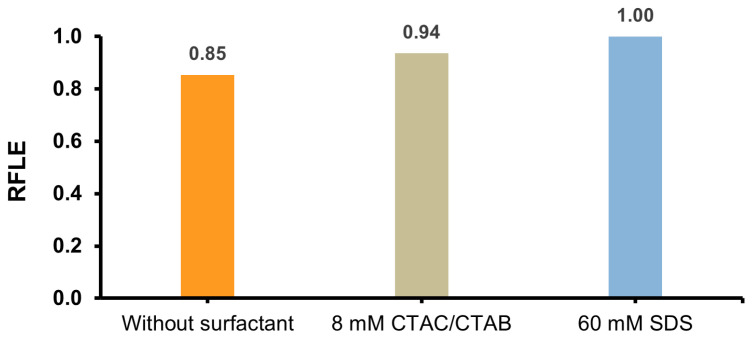
Comparison of the relative fluorescence efficiency (RFLE, normalised values) of the product obtained in the reaction of 2MeX with 0.1% H_2_O_2_ and 0.2 M NaOH in the presence of 8 mM CTAC/8 mM CTAB/60 mM SDS and without the addition of surfactant (RSD = 0.3–0.9%, *n* = 5). For details, see the Section 3.

The addition of both types of surfactants (cationic CTAC and CTAB and anionic SDS) causes slight changes in the FL emission spectra of 10Me-Aon (Figure 6 and Figure S11 in Supplementary Materials). The highest FL maximal intensity and relative efficiencies were obtained for the system containing SDS, while the lowest was without the surfactant. The spectra for both systems containing CTAC and CTAB overlap and are similar to the system without the addition of surfactants. Based on the measurements, it can be concluded that the addition of ionic surfactants does not significantly alter the fluorescent properties of the target emitter produced in the CL reaction, 10-methylacridan-9-one.

UV–Vis spectra of 2MeX substrate in an aqueous environment in the presence of investigated surfactants (SDS, CTAC, and CTAB) are presented in Figure S12 in Supplementary Materials. The graph indicates their relatively small impact on the parameters of electronic spectra, especially in the case of SDS. However, the anionic surfactant causes a slight hipsochromic shift of the spectra, which is not the case for CTAC/CTAB. The presence of the latter ones in the system (CTAC and especially CTAB) is manifested by distinct changes in the intensity (absorbance) of both characteristic bands (at ca. 260 nm and 367 nm), with no alternation of their spectral position.

### 2.4. Molecular Dynamics-Based Analysis

**Figure 7:**
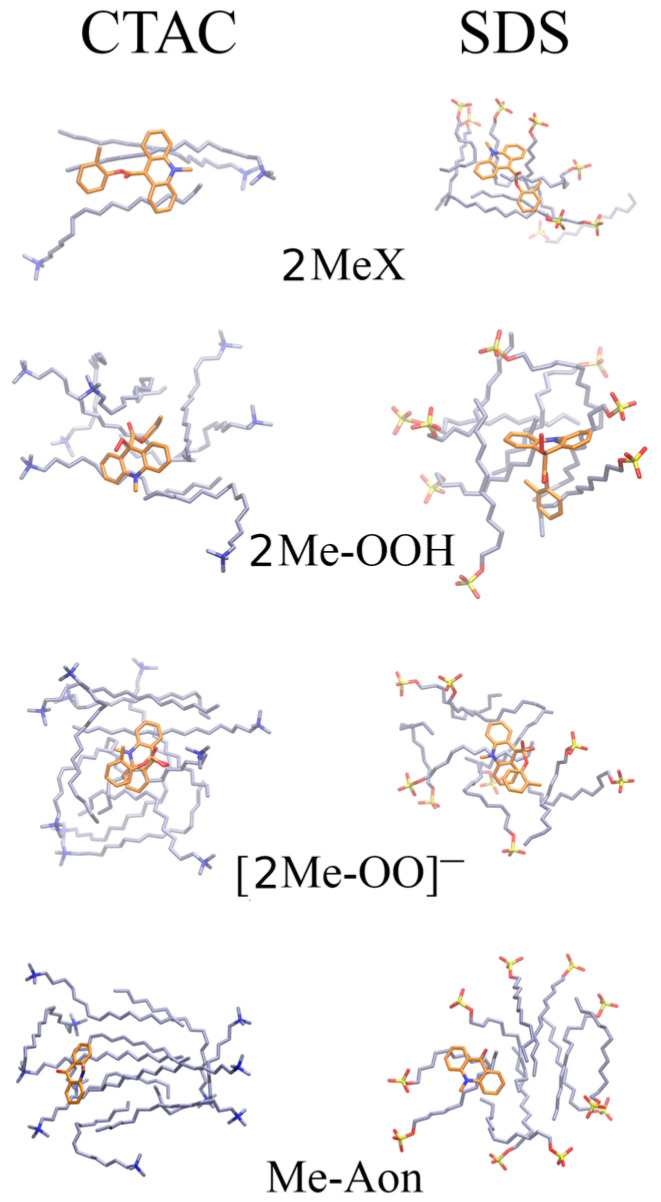
The structures obtained from the final frames of the MD simulations: carbon atoms of 2MeX, 2Me-OOH, [2Me-OO]^−^, and Me-Aon are marked in orange and those of surfactant molecules are marked in cyan.

To gain atomistic insights into potential interactions between 2MeX, 2Me-OOH, [2Me-OO]^−^, Me-Aon and surfactant molecules of two types (CTAC and SDS), 1 μs MD simulations were performed for each of the analysed molecules in the explicit solvent and counter ions in the presence of the 10 surfactant molecules. In all the simulated systems, already within the first nanoseconds of the MD simulation, surfactant molecules of both types established aggregates, which were bound to an analysed molecule. Interestingly, despite the differences between the four analysed molecules in terms of their charge (2MeX: +1, 2Me-OOH and Me-Aon: 0, [2Me-OO]^−^: −1), they all interacted with the surfactant aggregate predominantly by establishing hydrophobic contacts (Figure 7). The loss in the solvent-accessible surface area upon binding was very similar for both surfactants for all the analysed molecular systems, with slightly higher relative values for the solvent-accessible surface area for molecules interacting with the surfactant of the same charge sign (Table 4). This can be explained by a weak net effect of the electrostatic interactions observed in the MD simulations of the systems. However, the linear interaction energy (LIE) approach for calculating the binding free energy of interactions using a uniform dielectric constant of 80 does not reveal significant differences in terms of the electrostatic interactions (Table 5, Figure S13 in Supplementary Materials). This effect is also reflected in LIE component values for the charged 2MeX and [2Me-OO]^−^, for which the interactions driven by hydrophobic contacts with SDS and CTAC are stronger, respectively, than with the surfactant having the same sign of their net charge. The distributions of the distances between the molecules and the surfactant molecules in the interacting aggregates and the number of surfactant molecules observed at once in the aggregates are very similar for all the systems (Figures S14 and S15 in Supplementary Materials). Qualitative but significant differences observed in the strength of binding between 2MeX with CTAC and SDS could provide one potential explanation of the difference in the effect of these two surfactants on emissive properties observed experimentally: SDS binds 2MeX stronger than CTAC and thus limits its accessibility for the further reaction in the chemiluminogenic oxidation pathway.

### 2.5. Possible Side Reaction among Reagents

**Figure 8:**
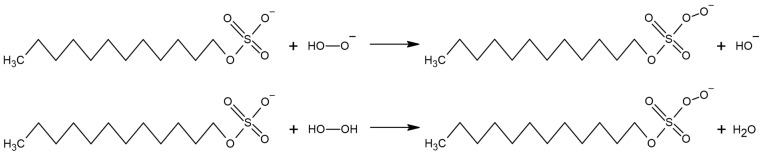
The putative transformation among the anionic surfactant under study, SDS, and the oxidiser used to trigger the emission of light from acridinium ester in an alkaline environment (H_2_O_2_/NaOH).

Investigating changes occurring during the generation of acridinium CL in the presence of various ionic surfactants and the possibility of other reaction routes, which could influence the changes observed in emission measurements, have also been considered. Namely, putative reactions that could occur between an oxidant, being one of the substrates necessary to trigger CL, namely, the OOH^−^ or H_2_O_2_ molecules and an anionic surfactant, i.e., SDS, were characterised (Figure 8).

Calculations at the level of DFT methods in gas and aqueous phases show that reactions of this type are not thermodynamically feasible due to the high values of the reaction’s Gibbs free energy in both cases (Table 6). Therefore, the possibility of generating changes in the CL process through side reactions among reagents, not including the chemiluminogenic substrate 2MeX, should be excluded.

## 3. Materials and Methods

### 3.1. Reagents and Working Solutions

All commercial reagents of analytical grade were used without additional purification. Hexadecyltrimethylammonium chloride (CTAC, ≥99%), hexadecyltrimethylammonium bromide (CTAB, ≥99%), sodium dodecyl sulphate (SDS, ≥99%) and anhydrous acetonitrile were purchased from Sigma Aldrich (St. Louis, MI, USA). Analytical weights (0.1 M) of hydrochloric acid, nitric acid(V) and sodium hydroxide were purchased from Chempur (Piekary Śląskie, Poland). Hydrogen peroxide 30% (p.a.) was purchased from Stanlab (Lublin, Poland). Ultrapure water (conductivity below 0.2 μS cm^−1^) (Beckman, Brea, CA, USA) was used to prepare all solutions. Aqueous solutions of surfactants for CL and FL measurements were prepared to obtain values before and above the CMC point, namely, 0.4 mM/0.8 mM and 4 mM/8 mM for CTAC and CTAB, and 10 mM/20 mM/40 mM/60 mM for SDS. Solutions of surfactants (8 mM CTAC, 8 mM CTAB, and 80 mM SDS) for conductometric measurements were prepared in an aqueous mixture containing 0.25 mM HCl and 0.5 mM HNO_3_.

Before CL and FL measurements, 0.1% H_2_O_2_ in 1 mM HNO_3_ and 0.2 M NaOH in water were prepared. The details concerning synthesis, chemical analyses and spectroscopic features (MS, NMR) of the chemiluminogenic substrate selected for this work, 10-methyl-9-[(2-methylphenoxy)carbonyl]acridinium trifluoromethanesulphonate (2MeX, Figure 2), have been described in our former work [19]. The 5 mM stock solution of 2MeX in anhydrous acetonitrile was kept in a freezer (253.15 K), and immediately before the experiments, the working solutions were prepared by its dilution to *c* = 0.1 μM with 1 mM HCl.

### 3.2. Conductometric Measurements

Conductometric measurements were performed using a microtitration unit (Cerko Lab System, Gdynia, Poland). The unit included a 5 mL syringe (Hamilton, Gdynia, Poland) and a CD-201 conductometric cell (Hydromet, Gliwice, Poland). The syringe was calibrated using a weight calibration method. The conductometric electrode was standardised using standards, specifically aqueous KCl solutions with conductivities of 84 and 200 μS cm^−1^ (Hamilton, Poland). The measurements were conducted at 298.15 ± 0.10 K and controlled using the Lauda E100 circulation thermostat. The reagents (CTAC, CTAB, and SDS) were dissolved directly in the solution containing 0.25 mM HCl and 0.5 mM HNO_3_. The experiment consisted of injecting, at 20 s intervals, 0.01 mL of the titrant solution (8 mM CTAC/8 mM CTAB/80 mM SDS) into the reaction cell, which initially contained a 5 mL mixture of 0.25 mM HCl and 0.5 mM HNO_3_. Two measurements were taken for each system, and the resulting data were analysed using the computer program Excel (Microsoft 365, Version 2405).

### 3.3. Measurements of Chemiluminescence

Measurements of CL were performed using a Fluoroskan Ascent FL microplate reader (Labsystems, Vantaa, Finland) with the detector tailored on maximal sensitivity (PMT voltage = 1000 mV). A 25 μL solution of 2MeX (*c* = 0.1 μM in 1 mM HCl) and 25 μL of surfactant (CTAC, CTAB, and SDS) solution in water were distributed onto a 96-well white polystyrene plate. After placing the plate in the apparatus, from the first dispenser, 50 μL of 0.1% H_2_O_2_ in 1 mM HNO_3_ was added to each well, and the plate was then shaken (10 s) at 600 rpm and incubated at 298.15 K for 2 min. The CL was induced and measured by adding 50 μL of 0.2 M NaOH to each well (*n* = 5 replicates for each concentration). The resolution of CL measurements was set at 20 ms for systems containing CTAC/CTAB and 300 ms for systems with SDS. The number of points was tailored to obtain a complete reaction profile in each case (100–1000 points). 

Each measurement of CL was repeated at least three times (*n* = 3–5) before the results were presented. The relative standard deviations (RSD) were calculated for each data group and are given in the captions denoting figures or tables. In all the CL data gathered for this work, the RSD factor was 0.3–6.7%. The results were processed using Excel software (Microsoft 365). 

### 3.4. Measurements of Fluorescence

The FL emission spectra of post-CL reaction mixtures were recorded at room temperature using a Cary Eclipse spectrofluorimeter (Varian, Palo Alto, CA, USA) with the detector’s sensitivity (PMT voltage) set at 600 mV and excitation/emission slits of 5 nm. The spectra were recorded using a standard 1 cm quartz cuvette in the 375–650 nm range, with the excitation wavelength set at 365 nm. The tested mixtures contained 1 mL of 2MeX (*c* = 50 μM) in 1 mM HCl, 2 mL of 0.1% H_2_O_2_ in 1 mM HNO_3_, 2 mL of 0.2 M NaOH and 1 mL of 8 mM CTAC/8 mM CTAB/60 mM SDS or UP water. The FL spectra were recorded just after the completion of the CL emission (*n* = 5 replicates for each system) and processed with the aim of the Excel software (Microsoft 365).

UV–Vis absorption spectra of 2MeX acridinium ester in 1 mM HCl with participation of surfactants were registered using a Lambda 40 Perkin-Elmer spectrophotometer and standard 1 cm quartz cuvettes. 

### 3.5. Molecular Dynamics-Based Analysis

The structures of 2MeX, 2Me-OOH, [2Me-OO]^−^, Me-Aon and both surfactant molecules CTA and SDS were first built in Avogadro [40,41] and further optimised in Gaussian16 [42] at DFT(B3LYP)/6-31G(d) [43,44,45,46] level of theory. Then, the RESP procedure [47] was applied to calculate the atomic partial point charges of these molecules compatible with the gaff force field [48] implemented in the AMBER20 package [49]. The missing bonded parameters for the equilibrium bond and angle values for [2Me-OO]^−^ were directly obtained from the Gaussian geometry optimisation, and the force constants were chosen to be characteristic for these types of bonds and angles to keep the geometry in the molecular dynamics (MD) simulation in agreement with the results from quantum chemical optimisation (30 kcal mol^−1^ Å^−2^ and 10 kcal mol^−1^ rad^−2^, respectively). 

MD simulations were performed in AMBER20 [49] for eight systems, each of which consisted of one molecule of either 2MeX or 2Me-OOH or [2Me-OO]^−^ or Me-Aon (chemical species appearing on the reaction path [19]) with 10 molecules of surfactant (CTAC or SDS) placed randomly. A truncated octahedron TIP3P periodic box of 15 Å water layer from the box’s border to solute was used to solvate the molecular systems. Cl^−^ or Na^+^ counter ions were used to neutralise the system’s charge. Two energy minimisation steps were performed: first, 500 steepest descent cycles and 103 conjugate gradient cycles with 100 kcal mol^−1^ Å^−2^ harmonic force restraints on the solute, and second, 3 × 10^3^ steepest descent cycles and 3 × 10^3^ conjugate gradient cycles without any restraints. Then, the system was heated from 0 to 300 K for 10 ps with harmonic force restraints of 100 kcal mol^−1^ Å^−2^ solute. Finally, the system was equilibrated at a constant temperature of 300 K and constant pressure of 10^5^ Pa for 100 ps. The productive MD run was performed in the same isothermal isobaric ensemble for 1 μs. The particle mesh Ewald method for treating electrostatics and the SHAKE algorithm for all the covalent bonds containing hydrogen atoms were applied. The obtained trajectories were analysed with the ccptraj module of AMBER20 [49]. Linear interaction energy (LIE) binding free energy calculations used the dielectric constant of 80. The visualisation and statistical analysis were performed in VMD [50] and R package [51], respectively.

### 3.6. The DFT Calculations

Optimised geometry of the studied compounds was performed utilising DFT [43] at the B3LYP [44,45] level of theory in conjunction with the 6-31G(d,p) [52] basis sets. The harmonic vibrational frequencies, characterising the stationary points, were evaluated to ensure that the obtained structures correspond to true minima on the potential energy surface. The calculations were achieved in the selected solvent (aqueous phase) by applying the polarisable continuum model (PCM) [53,54] with the Gaussian16 program [42], and the output files were visualised employing the ChemCraft program package [55]. 

The kinetics of CL decays were determined using a classical graphical method, and the thermodynamic parameters (enthalpy and Gibbs free energies) of the studied processes were calculated from the classical Hess equation [39].

## 4. Conclusions

Plate measurements of CL emission indicate interesting and different features of the acridinium ester-based aqueous system in the presence of ionic surfactants (CTAC, CTAB and SDS). This study generally aimed to explain, at the molecular level, the observed variabilities of the emission parameters of the chemiluminogenic substrate in the presence of the above surfactants. The selected substrate, 9-(2-methylphenoxy)carbonyl-10-methylacridinium triflate (2MeX), is characterised by good emission efficiency and stability in aqueous solutions, being employed in luminescence analysis as a CL indicator and fragment of CL labels. 

The CL parameters varied with the type of surfactant (cationic/anionic) and the pre-and post-micellar environment. Cationic surfactants exert a pronounced influence on the emission dynamics of CL, with a moderate impact on its efficiency. In contrast, the anionic surfactant exhibited an opposite effect, significantly slowing the emission dynamics while enhancing its efficiency. The CL kinetic constants and integral efficiencies in the presence of ionic surfactants were assessed before and after the CMC points and determined conductmetrically at comparable conditions. It has been observed that near and above the point of CMC, there is a significant (ca. 18-fold) increase in the emission rate constant for CTAC and a ca. 15-fold decrease in this parameter for SDS. 

Molecular dynamics analysis disclosed potential interactions of surfactant molecules with the intermediate products appearing during 2MeX chemiluminogenic transformations. The distances between the acridinium cation and the surfactant molecules and their number in the interacting aggregates were comparable for all the systems. At the same time, differences have been observed in the binding strength between 2MeX with CTAC and SDS, revealing that SDS molecules interact more strongly with the acridinium cation than CTAC ones.

Quantum chemical calculations at the DFT levels of theory have proven that the reaction of oxidant (e.g., H_2_O_2_ and its anionic form, OOH^−^) with an anionic surfactant (SDS) is not thermodynamically preferred, both in the aqueous and gaseous phases. Additionally, fluorescence emission measurements performed on the post-CL reaction mixtures excluded the significant influence of investigated surfactants on the emissive features of the final emitter, which is 10-methylacridan-9-one. 

In summary, research carried out employing experimental methods (CL and FL measurements) and computational ones (MD and DFT) indicate that interactions leading to significant changes in the observed emissive properties of the acridinium chemiluminogenic system occur at the reaction’s mechanism stage in the H_2_O_2_/NaOH aqueous environment, i.e., as a result of different interactions of the CL substrate (acridinium cation) and products of its oxidation, with cationic or anionic surfactant molecules. At the atomistic level, differences between CTAC and SDS interactions with the acridine-based molecular systems were detected due to the charge differences of the used surfactants.

The study has provided significant insights into the role of surfactants in the emissive features of acridinium chemiluminescence. The results can contribute to the rational optimisation of new chemiluminogenic systems, underscoring their practical implications. They also indicate that the acridinium ester may be an original and specific indicator of the surfactant type (cationic vs. anionic) and the system type (pre- or post-micellar).

## Figures and Tables

**Table 1:** CMC values for the tested surfactants determined conductometrically in the system of 0.25 mM HCl and 0.5 mM HNO_3_ at 298.15 K (relative standard deviations, RSDs, are below 0.5%).

Surfactant	CMC [mM]	
	Experimental Conditions	
	0.25 mM HCl + 0.50 mM HNO_3_	H_2_O [21]
CTAC	0.86	1.3
CTAB	0.68	0.9
SDS	7.34	8.1

**Table 2:** Experimental values obtained from measurements of chemiluminescence of investigated systems: maximum *I*_CL_ points, emission decay rate constants (*k*_CL_), and relative emission efficiencies (RCLE) (*c*_2MeX_ = 0.1 μM; PMT = 1000 mV; T = 298 K, RSD = 0.3–6.7%).

System		Maximal *I*_CL_ [RLU]	*k*_CL_ [s^−1^]	RCLE [RLU^2^]
No surfactant		379	0.179	2341
CTAC	0.4 mM	571	0.251	2427
	0.8 mM	1080	0.514	2520
	4.0 mM	4256	3.214	1433
	8.0 mM	4848	3.206	1615
CTAB	0.4 mM	653	0.277	2416
	0.8 mM	1691	0.897	2305
	4.0 mM	4732	3.422	1510
	8.0 mM	4838	3.405	1599
SDS	10 mM	205	0.130	2350
	20 mM	104	0.030	3320
	40 mM	91	0.015	6076
	60 mM	68	0.010	6398

**Table 3:** Summary of changes in the CL parameters of acridinium ester assessed in systems containing cationic (CTAC and CTAB) and anionic surfactants (SDS).

**Emission Process**	**Difference in CL Efficiency (%) Change in AUC Value (below/above CMC)**		
	**CTAC**	**CTAB**	**SDS**
CL	+6/−35	+1/−34	+21/+166
FL (>CMC)	+9	+9	+15
**Emission Process**	**The Difference in CL Dynamics (%) Change in *k*_CL_ Value (below/above CMC)**		
	**CTAC**	**CTAB**	**SDS**
CL	+114/+1697	+228/+1807	−55/−93

**Table 4:** Relative solvent-accessible surface area (as a ratio between the one observed in the MD simulation and the maximum value corresponding to the unbound molecule).

Relative Solvent-Accessible Surface Area				
Surfactant	2MeX	2Me-OOH	[2Me-OO]^−^	Me-Aon
CTAC	0.71 ± 0.12	0.65 ± 0.14	0.60 ± 0.13	0.54 ± 0.13
SDS	0.65 ± 0.14	0.63 ± 0.13	0.73 ± 0.13	0.59 ± 0.15

**Table 5:** LIE free energy components (electrostatic (Ele) and van der Waals (VDW), in kcal mol^−1^) and total energy of binding between the entities appearing on the chemiluminescence path of 2MeX and surfactant molecules.

Surfactant	2MeX			2Me-OOH			[2Me-OO]^−^			Me-Aon		
	Ele	VDW	Total	Ele	VDW	Total	Ele	VDW	Total	Ele	VDW	Total
CTAC	0.02	−1.76	−1.74	0.00	−1.97	−1.97	−0.06	−2.01	−2.07	0.00	−1.69	−1.69
SDS	−0.06	−1.98	−2.04	−0.01	−1.99	−2.00	0.02	−1.62	−1.60	0.00	−1.58	−1.58

**Table 6:** Thermodynamic parameters (enthalpy (Δ_r,298_*H*^0^) and Gibbs free energy in gas and aqueous phase (Δ_r,298_*G*^0^), in kcal mol^−1^) in the putative reaction between SDS surfactant and hydroperoxide anion (OOH^−^) or hydrogen peroxide (Figure 8). For details, see the Section 3.

Gas Phase		Aqueous Phase	
Δ_r,298_*H*^0^	Δ_r,298_*G*^0^	Δ_r,298_*H*^0^	Δ_r,298_*G*^0^
**reaction between SDS and OOH^−^**			
55.1	57.6	49.9	52.4
**reaction between SDS and H_2_O_2_**			
28.3	29.7	30.4	31.8

## Data Availability

Data are contained within the article and Supplementary Materials.

## Supplementary Materials

The following supporting information can be downloaded at: https://www.mdpi.com/article/10.3390/molecules29163736/s1, Figure S1: The electrical conductivity (χ) as a function of the surfactant concentration recorded in the 0.25 mM HCl and 0.5 mM HNO_3_ system, at 298 K; Figure S2: CL kinetic profiles for systems 2MeX (0.1 μM)/H_2_O_2_ (0.1%)/NaOH (0.2 M) containing various concentrations of cationic surfactants (0.4 mM and 0.8 mM CTAC and CTAB); Figure S3: CL kinetic profiles for systems 2MeX (0.1 μM)/H_2_O_2_ (0.1%)/NaOH (0.2 M) containing various concentrations of cationic surfactants (4 mM and 8 mM CTAC and CTAB); Figure S4: CL kinetic profiles for systems 2MeX (0.1 μM)/H_2_O_2_ (0.1%)/NaOH (0.2 M) containing various concentrations of anionic surfactant (10 mM and 20 mM SDS); Figure S5: CL kinetic profiles for systems 2MeX (0.1 μM)/H_2_O_2_ (0.1%)/NaOH (0.2 M) containing various concentrations of anionic surfactant (40 mM and 60 mM SDS); Figure S6: Dependence of ln(*I*_CL_) as a function of time for the 2MeX/H_2_O_2_/NaOH system with no surfactant; Figure S7: Dependence of ln(*I*_CL_) as a function of time for systems containing various concentrations of CTAC; Figure S8: Dependence of ln(*I*_CL_) as a function of time for systems containing various concentrations of CTAB; Figure S9: Dependence of ln(*I*_CL_) as a function of time for systems containing various concentrations of SDS; Figure S10: Correlation between the emission decay rate constants (*k*_CL_) and the maximum values of emission (*I*_CL_^max^), determined from the CL time–response profiles; Figure S11: FL emission spectra of 10-methylacridan-9-one, the main product obtained in the reaction of 2MeX with 0.1% H_2_O_2_ and 0.2 M NaOH in the presence of 8 mM CTAC/8 mM CTAB/60 mM SDS and without the addition of surfactant; Figure S12: Electronic absorption spectra (UV–Vis) for the acridinium chemiluminescence emitter 2MeX in the presence of the investigated ionic surfactants near the CMC point; Figure S13: Density of probability for free binding energy values corresponding to the interactions with surfactant molecules observed in the MD simulations: CTAC (in black) and SDS (in red); Figure S14: Density of probability for the distances between the molecules and surfactant molecules observed in the MD simulations: CTAC (in black) and SDS (in red); Figure S15: Density of probability for the number of surfactant molecules within 15 Å of the analysed acridine species, observed in the MD simulations: CTAC (in black) and SDS (in red).
