# Supplementary material for: Why Do Ionic Surfactants Significantly Alter the Chemiluminogenic Properties of Acridinium Salt?
Source: Molecules. 2024 Aug 7;29(16):3736. doi: 10.3390/molecules29163736 (PMC11356875; doi:10.3390/molecules29163736)
Supplement: Supplementary file 1 [file molecules-29-03736-s001.zip › molecules-3117091-supplementary.pdf]

Supplementary Materials

Why do ionic surfactants significantly alter the chemiluminogenic properties of acridinium salt?

Magdalena Mańkowska, Karol Krzymiński *, Dariusz Wyrzykowski, Beata Zadykowicz, and Sergey A. Samsonov*

Faculty of Chemistry, University of Gdańsk, Wita Stwosza 63, 80-308 Gdańsk, Poland

** Correspondence: karol.krzyminski@ug.edu.pl (K.K.), sergey.samsonov@ug.edu.pl (S.A.S.)*

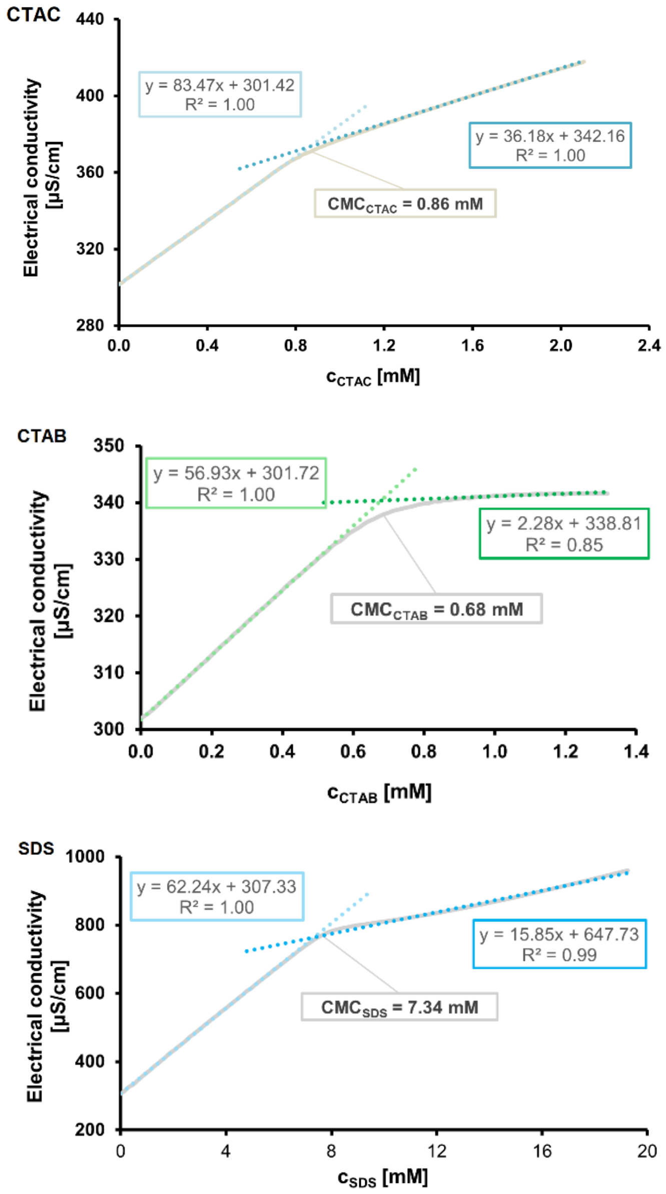

Figure S1. The electrical conductivity (χ) as a function of the surfactant concentration (CTAC (top graph), CTAB (middle graph), SDS (bottom graph)) recorded in the 0.25 mM HCl and 0.5 mM HNO_3 system, at 298 K.

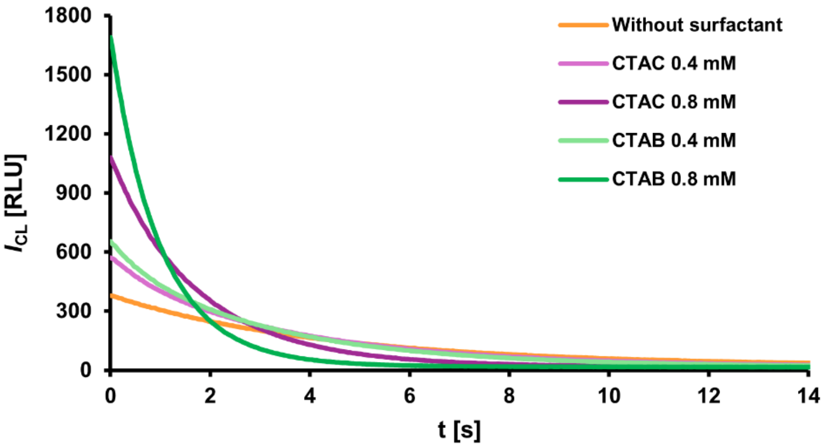

Figure S2. Chemiluminescence kinetic profiles for systems 2MeX (0.1 μM)/H_2O_2 (0.1%)/NaOH (0.2 M) containing various concentrations of cationic surfactants (0.4 mM and 0.8 mM CTAC and CTAB) (sensitivity: PMT = 1000 mV; 20 ms/pt; T = 298 K).

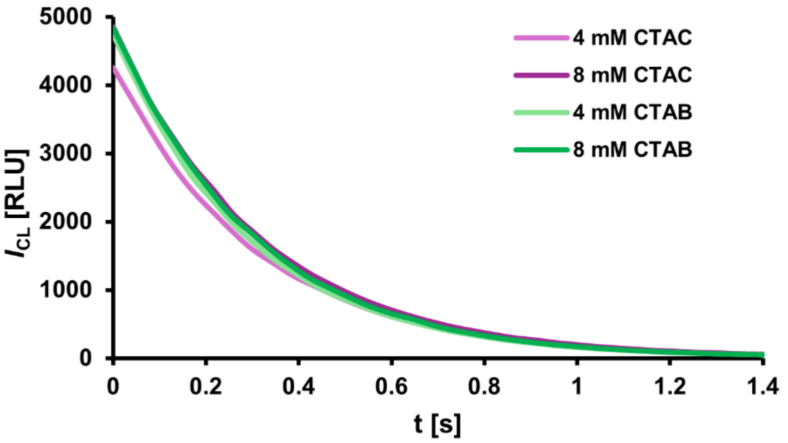

Figure S3. Chemiluminescence kinetic profiles for systems 2MeX (0.1 μM)/H_2O_2 (0.1%)/NaOH (0.2 M) containing various concentrations of cationic surfactants (4 mM and 8 mM CTAC and CTAB) (sensitivity: PMT = 1000 mV; 20 ms/pt; T = 298 K).

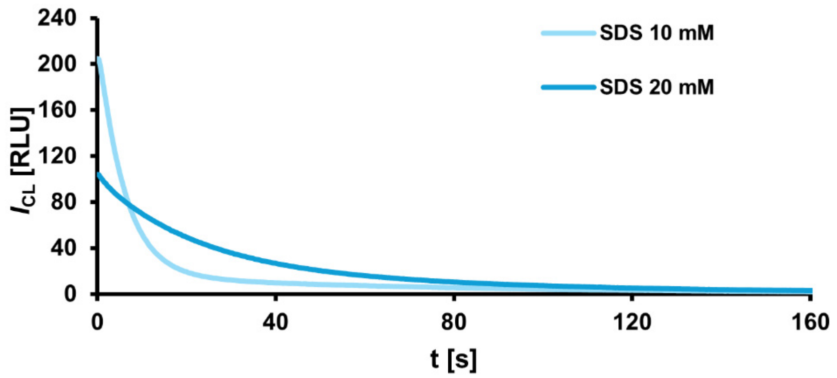

Figure S4. Chemiluminescence kinetic profiles for systems 2MeX (0.1 μM)/H_2O_2 (0.1%)/NaOH (0.2 M) containing various concentrations of anionic surfactant (10 mM and 20 mM SDS) (sensitivity: PMT = 1000 mV; 20 ms/pt; T = 298 K).

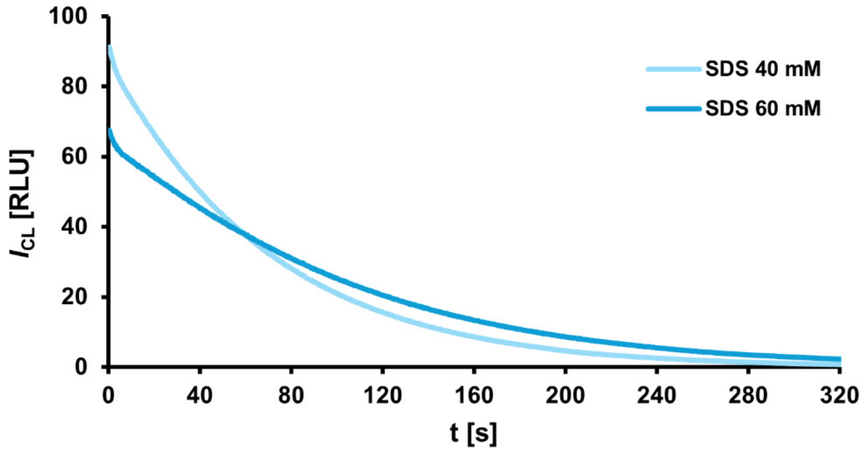

Figure S5. Chemiluminescence kinetic profiles for systems 2MeX (0.1 μM)/H_2O_2 (0.1%)/NaOH (0.2 M) containing various concentrations of anionic surfactant (40 mM and 60 mM SDS) (sensitivity: PMT = 1000 mV; 20 ms/pt; T = 298 K).

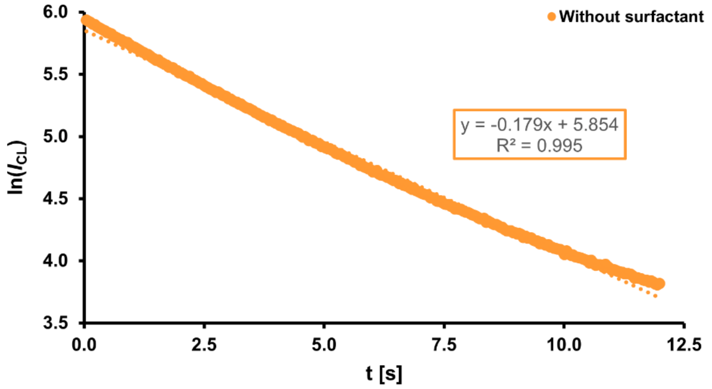

Figure S6. Dependence of ln(I_{CL}) as a function of time for the 2MeX/H_2O_2/NaOH system with no surfactant (c_{2MeX} = 0.1 μM; 20 ms/pt; PMT = 1000 mV; T = 298 K).

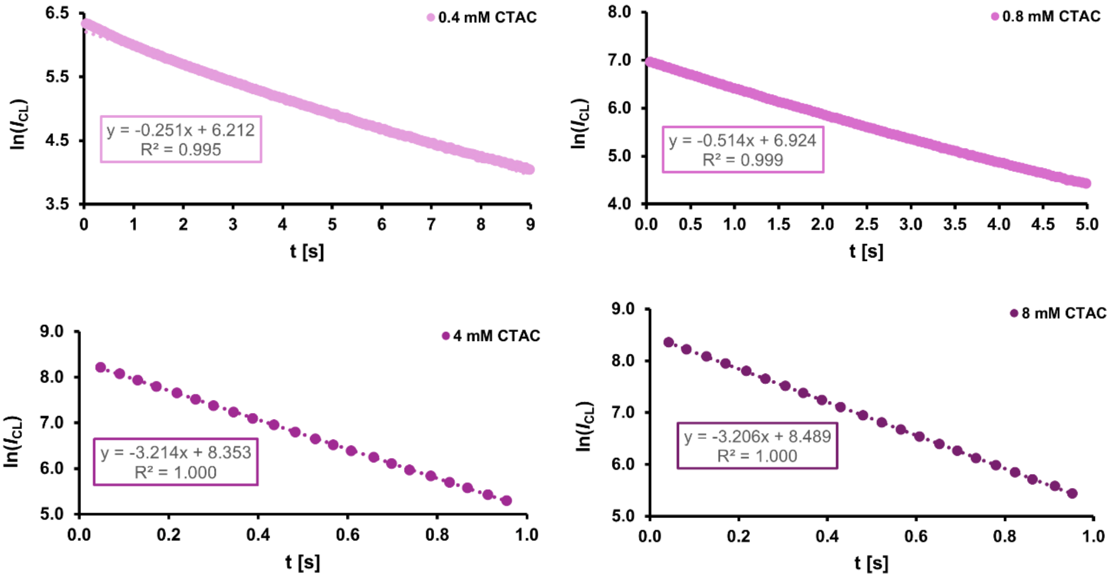

Figure S7. Dependence of ln(I_{CL}) as a function of time for systems containing various concentrations of CTAC (c_{2MeX} = 0.1 μM; 20 ms/pt; PMT = 1000 mV; T = 298K).

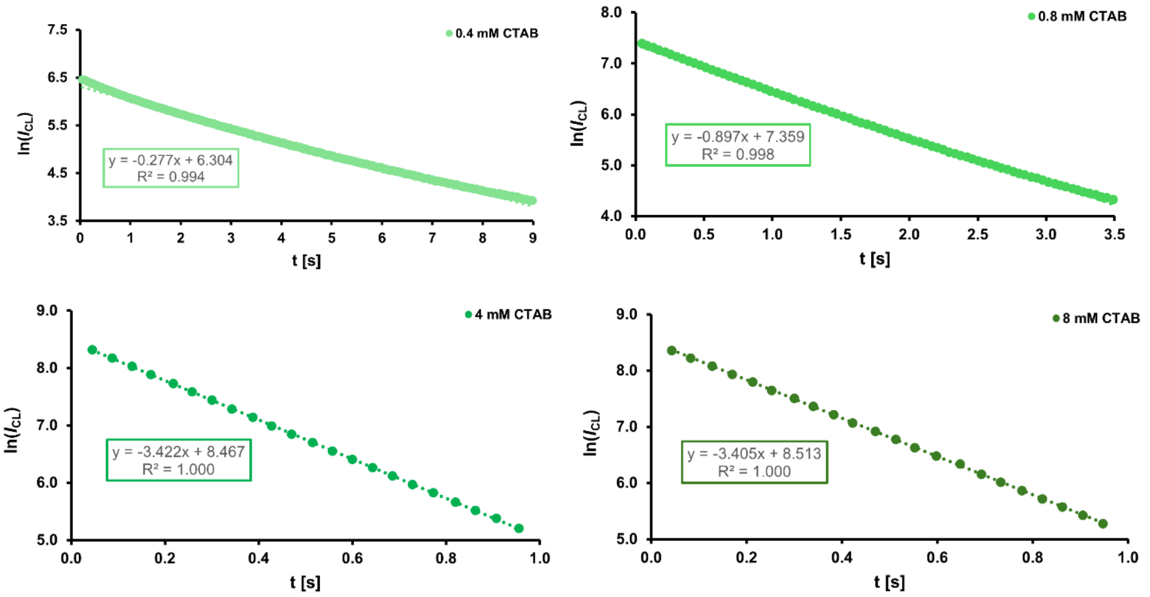

Figure S8. Dependence of ln(I_{CL}) as a function of time for systems containing various concentrations of CTAB (c_{2MeX} = 0.1 μM; 20 ms/pt; PMT = 1000 mV; T = 298K).

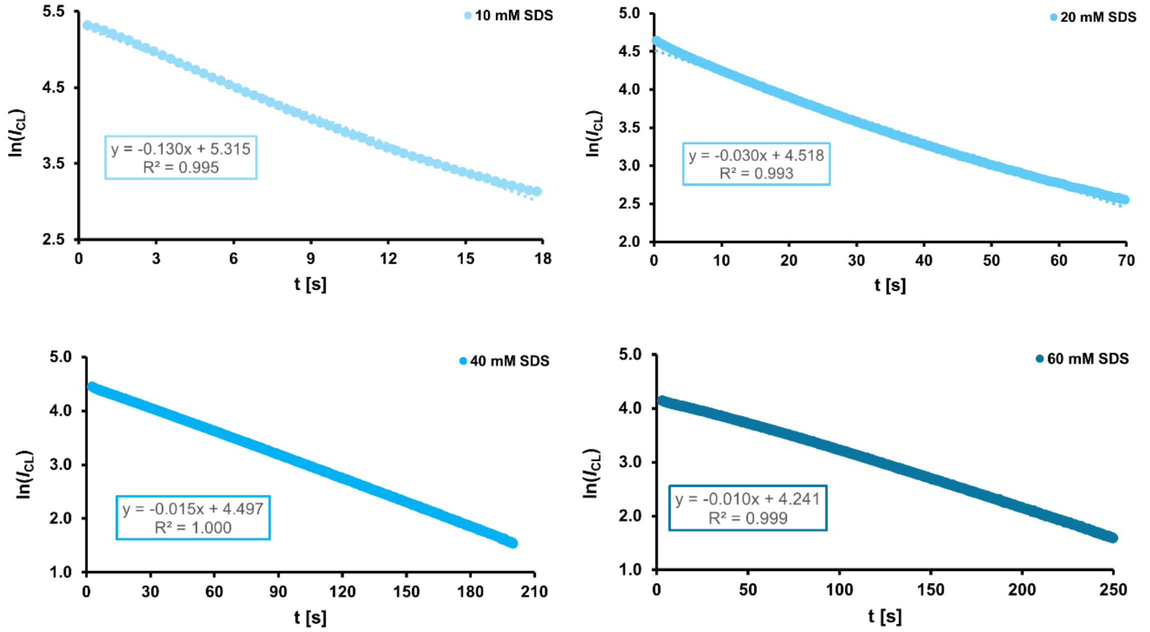

Figure S9. Dependence of ln(I_{CL}) as a function of time for systems containing various concentrations of SDS (c_{2MeX} = 0.1 μM; 20 ms/pt; PMT = 1000 mV; T = 298K).

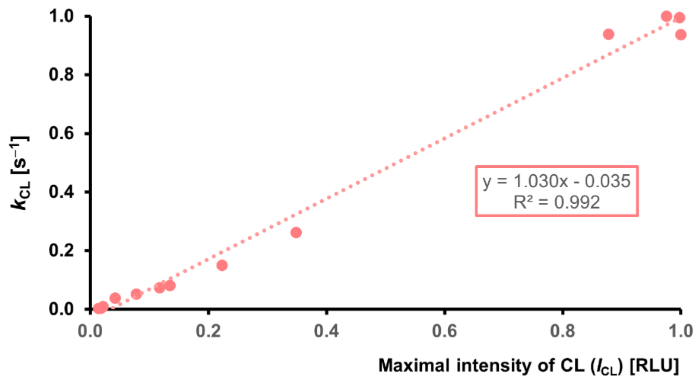

Figure S10. Correlation between the emission decay rate constants (k_{CL}) and the maximum values of emission (I_{CL}^{max}), determined from the CL time-response profiles.

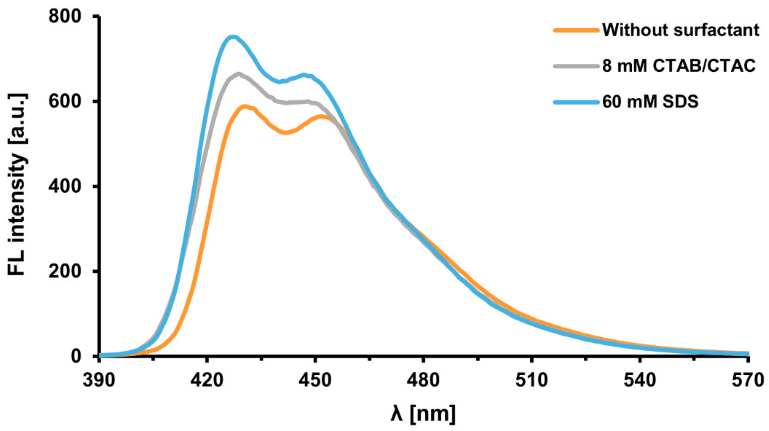

Figure S11. Fluorescence emission spectra of 10-methylacridan-9-one – the main product obtained in the reaction of 2MeX with 0.1% H_2O_2 and 0.2 M NaOH in the presence of 8 mM CTAC/8 mM CTAB/60 mM SDS and without the addition of surfactant; (c_{2MeX} = 50 µM; λ_{ex} = 365 nm; PMT = 600 mV; T = 298 K).

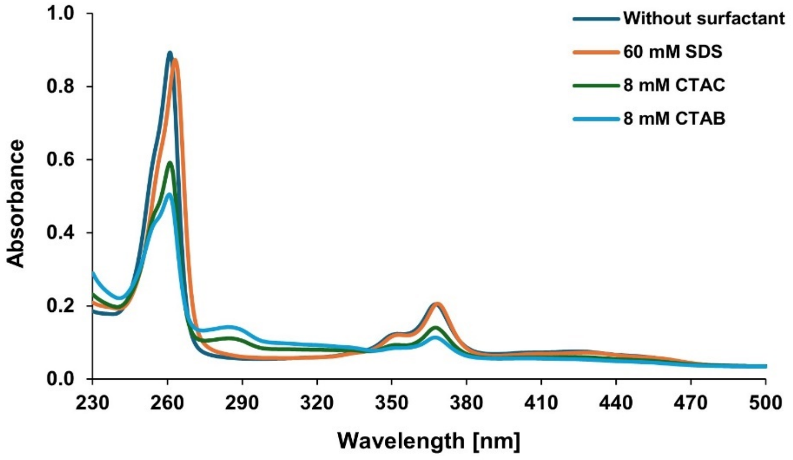

Figure S12. Electronic absorption spectra (UV-Vis) for the acridinium chemiluminescence emitter 2MeX in the presence of the investigated ionic surfactants near the CMC point. The Materials and Methods section provides detailed information on the experiment.

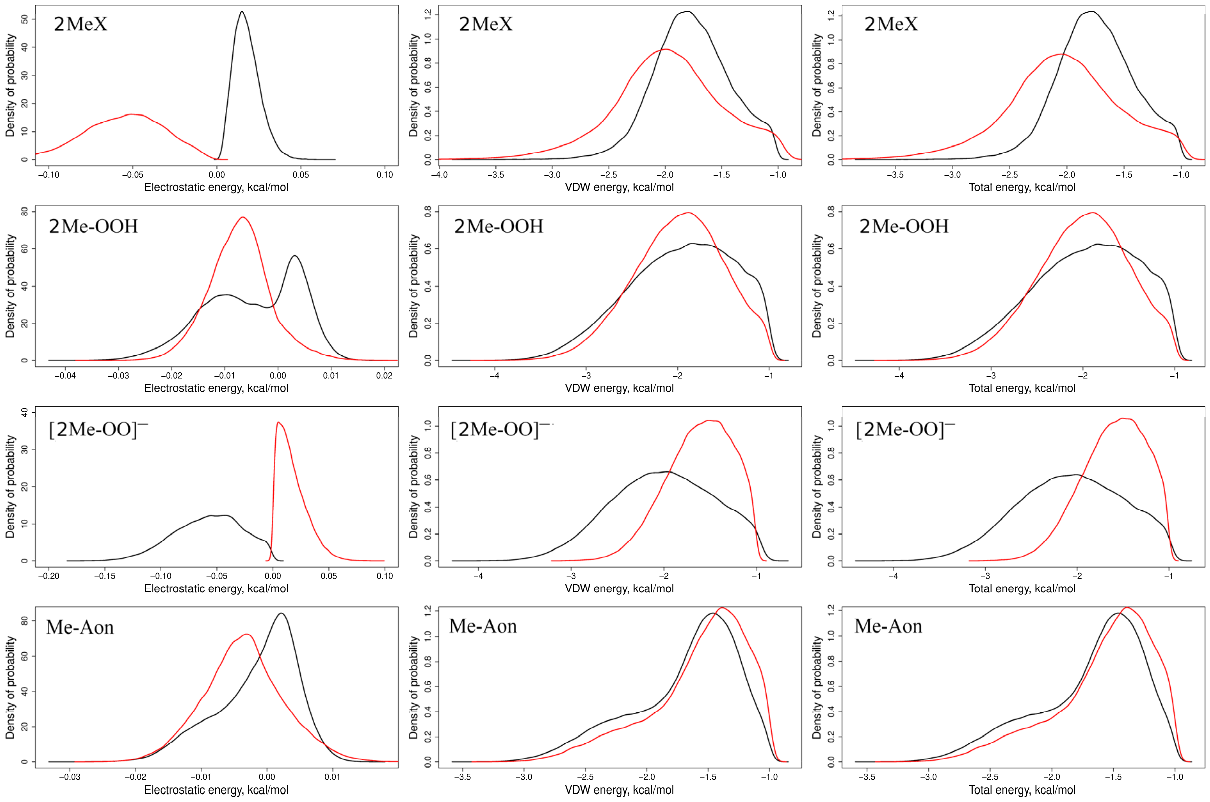

Figure S13. Density of probability for free binding energy values corresponding to the interactions with surfactant molecules observed in the MD simulations: CTAC (in black) and SDS (in red).

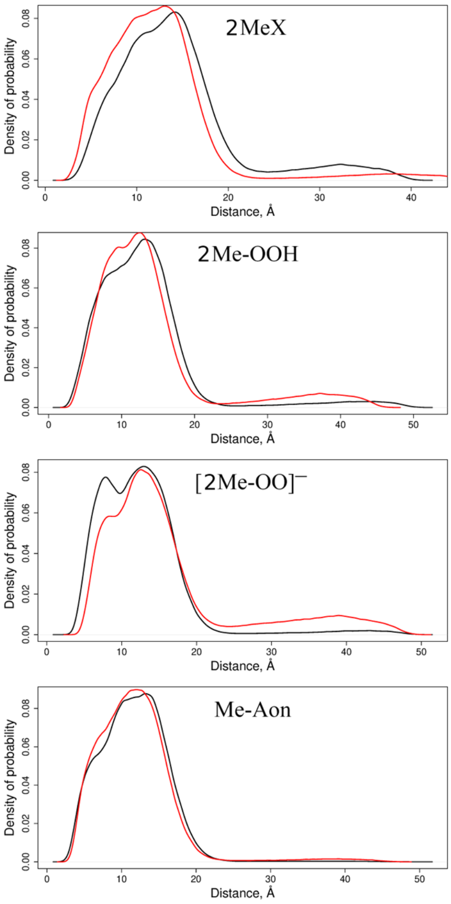

Figure S14. Density of probability for the distances between the molecules and surfactant molecules observed in the MD simulations: CTAC (in black) and SDS (in red).

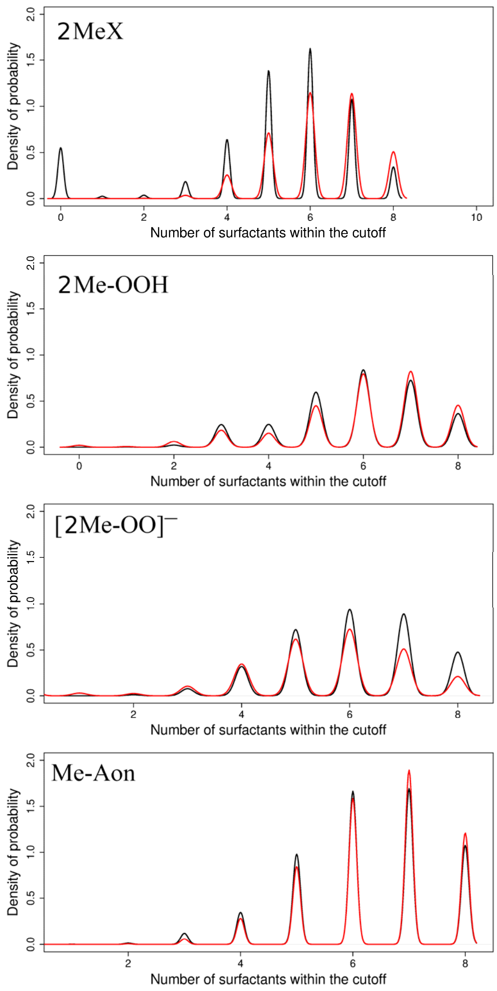

Figure S15. Density of probability for the number of surfactant molecules within 15 Å of the analyzed acridine species, observed in the MD simulations: CTAC (in black) and SDS (in red).
